# The triple variable index combines information generated over time from common monitoring variables to identify patients expressing distinct patterns of intraoperative physiology

**DOI:** 10.1186/s12874-019-0660-9

**Published:** 2019-01-14

**Authors:** Michael P. Schnetz, Harry S. Hochheiser, David J. Danks, Douglas P. Landsittel, Keith M. Vogt, James W. Ibinson, Steven L. Whitehurst, Sean P. McDermott, Melissa Giraldo Duque, Ata M. Kaynar

**Affiliations:** 1 Department of Anesthesiology, University of Pittsburgh, 3550 Terrace Street, Pittsburgh, PA 15261 USA; 2 Department of Biomedical Informatics, University of Pittsburgh, 5607 Baum Boulevard, Pittsburgh, PA 15206 USA; 3 Departments of Philosophy and Psychology, Carnegie Mellon University, 5000 Forbes Avenue, Pittsburgh, PA 15213 USA; 4 Department of Critical Care Medicine, University of Pittsburgh, 3550 Terrace Street, Pittsburgh, PA 15261 USA

**Keywords:** Triple variable index, Triple low state, Mean arterial pressure, Bispectral index, Minimum alveolar concentration, K-means clustering, Unsupervised machine learning, Perioperative medicine, Intraoperative monitoring, Postoperative death

## Abstract

**Background:**

Mean arterial pressure (MAP), bispectral index (BIS), and minimum alveolar concentration (MAC) represent valuable, yet dynamic intraoperative monitoring variables. They provide information related to poor outcomes when considered together, however their collective behavior across time has not been characterized.

**Methods:**

We have developed the Triple Variable Index (TVI), a composite variable representing the sum of z-scores from MAP, BIS, and MAC values that occur together during surgery. We generated a TVI expression profile, defined as the sequential TVI values expressed across time, for each surgery where concurrent MAP, BIS, and MAC monitoring occurred in an adult patient (≥18 years) at the University of Pittsburgh Medical Center between January and July 2014 (*n* = 5296). Patterns of TVI expression were identified using k-means clustering and compared across numerous patient, procedure, and outcome characteristics. TVI and the triple low state were compared as prediction models for 30-day postoperative mortality.

**Results:**

The median frequency MAP, BIS, and MAC were recorded was one measurement every 3, 5, and 5 min. Three expression patterns were identified: elevated, mixed, and depressed. The elevated pattern displayed the highest average MAP, BIS, and MAC values (86.5 mmHg, 45.3, and 0.98, respectively), while the depressed pattern displayed the lowest values (76.6 mmHg, 38.0, 0.66). Patterns (elevated, mixed, depressed) were distinct across the following characteristics: average patient age (52, 53, 54 years), American Society of Anesthesiologists Physical Status 4 (6.7, 16.1, 27.3%) and 5 (0.1, 0.6, 1.6%) categories, cardiac (2.2, 6.5, 16.1%) and emergent (5.8, 10.5, 12.8%) surgery, cardiopulmonary bypass use (0.3, 2.6, 9.8%), intraoperative medication administration including etomidate (3.0, 7.3, 12.6%), hydromorphone (47.6, 26.3, 25.2%), ketamine (11.2, 4.6, 3.0%), dexmedetomidine (18.4, 16.6, 13.6%), phenylephrine (74.0, 74.8, 83.0), epinephrine (2.0, 6.0, 18.0%), norepinephrine (2.4, 7.5, 21.2%), vasopressin (3.4, 7.6, 21.0%), succinylcholine (74.0, 69.0, 61.9%), intraoperative hypotension (28.8, 33.0, 52.3%) and the triple low state (9.4, 30.3, 80.0%) exposure, and 30-day postoperative mortality (0.8, 2.7, 5.6%). TVI was a better predictor of patients that died or survived in the 30 days following surgery compared to cumulative triple low state exposure (AUC 0.68 versus 0.62, *p* < 0.05).

**Conclusions:**

Surgeries that share similar patterns of TVI expression display distinct patient, procedure, and outcome characteristics.

**Electronic supplementary material:**

The online version of this article (10.1186/s12874-019-0660-9) contains supplementary material, which is available to authorized users.

## Background

Providing effective and safe care for patients during surgery requires high frequency, multi-organ system physiologic monitoring. Intraoperative events (e.g. hypotension and hypoxemia) often occur rapidly, and clinicians rely on intraoperative monitoring information to promptly diagnose and treat such events. Mean arterial blood pressure (MAP), bispectral index (BIS), and minimum alveolar concentration (MAC) are monitored to maintain safe blood pressure and anesthetic levels during surgery. The information they provide helps clinicians avoid serious complications related to intraoperative management. Intraoperative hypotension, for example, increases a patient’s risk of acute kidney injury, myocardial infarction and even death [1–6], while intraoperative awareness increases a patient’s risk of experiencing anxiety and post-traumatic stress disorder after surgery [7].

Recent studies have demonstrated that MAP, BIS, and MAC data, when combined, provide information beyond that used to assess blood pressure and anesthetic depth levels. The triple low state, defined as a MAP < 75 mmHg, BIS < 45, and MAC < 0.8, informs a patient’s risk of postoperative death and increased length of hospitalization [8–10]. Although a valuable tool to capture risk information, the triple low state is ill-suited as a model for the combined behavior of these complex, dynamic variables. Specifically, the triple low state 1) is defined by variable thresholds and therefore represents only a subset of possible MAP, BIS, and MAC combinations, 2) has been studied in select patient populations (e.g. non-cardiac [8, 9], patients at high risk of intraoperative awareness and recall [10]), 3) lacks a time component and cannot explain how variable combinations occur across the intraoperative period. New approaches that overcome these limitations are needed to broaden our understanding of MAP, BIS, and MAC combinations and their relationship to key patient, procedure, and outcome characteristics. Here, we define a new index called the Triple Variable Index (TVI) that combines equally-weighted MAP, BIS, and MAC values into a single composite variable representing the underlying MAP, BIS, and MAC combination at any particular moment. TVI values can be mapped across time and for any patient in whom requisite data are available including those undergoing cardiac surgery.

The aims of our study were three-fold: 1) generate TVI data for all adult patients that underwent surgery within our study period and in whom MAP, BIS, and MAC data were available for analysis, 2) plot TVI values in a temporal manner across the intraoperative period, and 3) identify common patterns of TVI “expression” and their associated patient, procedure, and outcome characteristics. Our index represents a novel, data-driven approach that incorporates all available MAP, BIS, and MAC information to improve our understanding of the global nature of these variables during surgery in ways not possible using the triple low state model.

## Methods

### Study population

Surgeries that took place between January 1, 2014 and July 31, 2014 at the University of Pittsburgh Medical Center (UPMC) Presbyterian and Montefiore hospitals were evaluated for study inclusion. Surgeries were included in the study if 1) the patient undergoing surgery was at least 18 years old and 2) MAP, BIS, and end tidal inhaled anesthetic concentrations were concurrently monitored and recorded in the electronic anesthesia record system. Surgeries that did not meet these inclusion criteria were excluded from analysis. The Institutional Review Board at the University of Pittsburgh approved this retrospective study (PRO15060130) and waived the informed consent requirement.

### Data extraction, MAC calculation, and artifact removal

For each study surgery, the following data were extracted from the electronic record systems at UPMC: MAP, BIS (Quatro Sensor, Covidien, Minneapolis, MN), inhaled anesthetic concentrations (isoflurane, desflurane, sevoflurane, nitrous oxide), patient age, gender, medical record number, assigned American Society of Anesthesiology (ASA) Physical Status, data of surgery, surgical specialty, type of procedure, procedure length in minutes, use of cardiopulmonary bypass, and date of postoperative death. All available MAP, BIS, and inhaled concentrations were used in our analysis. These variables were not recorded at a uniform frequency at all times, and therefore their recording frequencies are reported as results. Data were not assumed at any times where measurements were not actually measured and recorded. Mortality data were collected from the United States Social Security Death Index on August 22, 2016. All administered doses of each of the following intravenous anesthetics/adjuncts, opioid, vasopressor and muscle relaxant medications were extracted: midazolam, propofol, etomidate, fentanyl, remifentanil, hydromorphone, morphine, ketamine, dexmedetomidine, ephedrine, phenylephrine, epinephrine, norepinephrine, vasopressin, succinylcholine, rocuronium, and cisatracurium.

Both noninvasive and arterial line MAP values were collected; when both were present, arterial line measurements were used. A total MAC value was calculated at each time an inhaled agent was recorded. The total MAC value was calculated by summing individual agents’ standard 1 MAC equivalents: isoflurane = 1.17%, desflurane = 6.6%, sevoflurane = 1.8%, and nitrous oxide = 105% [11]. For our purposes, the term ‘MAC’ refers to the summed MAC values representing all inhaled anesthetics at a given time. MAC values were not age-adjusted because we sought to evaluate TVI expression that represents unadjusted MAP, BIS, and MAC values as they occur in patients. Inhaled anesthetics were considered to be used any time a summed MAC value was greater than 0.001; values less than this were considered clinically negligible.

Extreme values, reflecting artifacts in the data, were removed. The limits were defined as MAP values greater than 250 or less than 10 mmHg, MAC values greater than 3, and BIS values greater than 100 or less than 1. Effects on variable measurement such as movement of the arterial blood pressure transducer and electrosurgical interference were not explicitly identified in the anesthesia electronic record system. Unless such effects resulted in measurements outside of the limits noted above, they were not accounted for in our analysis. A BIS value of zero was considered artifact because this value occurred at the very beginning and end of the monitoring period suggesting a common artifact associated with initiating and discontinuing BIS monitoring.

### TVI generation and profile visualization

Following MAC value calculation and artifact removal, MAP, MAC, and BIS data were normalized using a z-score. These variables demonstrated an approximately normal distribution as assessed with histogram analysis. A z-score was calculated for each individual measurement relative to the total population of values for that given variable. MAP, MAC, and BIS data were not measured and/or recorded at the same frequency, thus were aggregated using a non-overlapping, sliding window approach [12]. For each variable, an average value was calculated within sequential windows, each defined as five consecutive measurements where MAP, BIS, or inhaled anesthetics were monitored as single variables or in combination, starting at the beginning of the monitoring period. In cases where five measurements were not available at the end of the monitoring period, an average value was calculated for the last window using the remaining measurements. A TVI value was generated for each window by summing z-scores of MAP, MAC, and BIS variables. TVI values were not generated, by definition, for windows lacking data for any one of the MAP, MAC, or BIS variables. At least 1 TVI value was generated for all study surgeries. Inhaled anesthetic administration during cardiopulmonary bypass is recorded by a perfusionist at UPMC and not captured by the electronic anesthesia record system. TVI values were not generated for these periods due to a lack of available MAC data.

A TVI profile, representing the total TVI values sequentially mapped across the intraoperative period, was generated for each study surgery. Profiles were aligned along their window sequence in the same plot to visualize and compare together. The number of TVI values generated for each profile varied, and the color white in these plots represents a lack of TVI data because 1) surgery was not taking place, or 2) surgery was occurring but data from one or more of the TVI variables (MAP, BIS, MAC) were absence. All TVI values calculated and plotted were positive (represented as red) or negative (represented as blue) values. TVI values of “0” did not exist in our dataset.

### Identification of TVI expression patterns using k-means clustering

An unsupervised machine learning algorithm called k-means clustering [13] was performed to identify groups of profiles that shared a similar pattern of TVI expression across time. The k-means algorithm works by iteratively selecting and segregating TVI profiles into clusters so the sum of squares between the data points within clusters are minimized. We chose k-means clustering because it represents a common, conceptually straightforward approach that has been applied to grouping problems across several fields of study [14]. The number of clusters used to segregate the dataset is user defined and must be identified a priori. We performed multiple analyses using 2, 3, 4, and 5 clusters; each with 10 random starts and 100 maximum iterations. The clusters identified in the 3-cluster analysis were selected for further analysis because they differentiated profiles according to TVI expression and associated 30-day mortality while maximizing the number of profiles in each cluster.

### Characterization of TVI expression patterns

Patient and procedure characteristics, MAP, BIS, MAC, and TVI values and their Pearson correlations, intraoperative medication administration, intraoperative hypotension, triple low state exposure, and postoperative mortality were compared between clusters. Means and medians were calculated for continuous variables, proportions were calculated for categorical variables. Means were generated for continuous variables demonstrating an approximately normal distribution according to histogram analysis. Otherwise, a median was generated. Standard deviation, 1st and 3rd quartiles, and violin plots were generated to demonstrate variable distributions. Ninety-five percent confidence intervals were calculated for individual mean, median, and proportion statistics. A bootstrapping approach was used to generate a 95% confidence interval for a median statistic. For a given variable, 10,000 samples were generated each containing 1,000,000 randomly selected data values. The median was calculated for each sample. The 2.5 and 97.5 quantiles of this population of medians defined the 95% confidence interval for the variable’s median statistic.

MAP, BIS, MAC and TVI values were compared across time during the intraoperative period using a generalized additive model with cubic regression splines and Gaussian distributions (geom_smooth function in ggplot2 package available in RStudio). To standardize the x-axis in these plots, the timestamp associated with each MAP, BIS, and MAC value was converted to a proportion of the total monitoring period defined by the first and last MAP, BIS, and/or inhaled anesthetic concentration measurement recorded during the surgery. For example, if 60 min separated the first and last measurements recorded for a study surgery, a measurement recorded at minute six would be associated with a proportion value of 0.10. For TVI values, proportions were calculated based on a surgery’s total number of profile windows. All MAP, BIS, MAC, and TVI values were associated with a proportion between 0.0 and 1.0.

A profile was identified as having experienced intraoperative hypotension if any recorded MAP level was less than 55 mmHg. A profile was identified as having experienced the triple low state if any profile window met established triple low criteria: a concurrent MAP < 75 mmHg, BIS < 45, and MAC < 0.8 [9]. Although the triple low state has been most commonly associated with 30-day postoperative mortality [8–10], Kertai and colleagues demonstrated mortality differences for two years following surgery between triple low state exposure groups in their unadjusted analyses. In addition to 30-day postoperative mortality, mortality that occurred within postoperative days 31–365 and 366–730 was compared between clusters. A sensitivity analysis was conducted to recalculate mortality proportions because our study did not exclude profiles representing repeat surgery. The proportion of individual patients, rather than individual profiles, associated with each mortality group was calculated for each cluster.

To further examine the relationship between TVI expression and death following surgery, TVI expression was evaluated as a prediction model for 30-day postoperative mortality and compared to a triple low state model. The risk of 30-day postoperative mortality increases as triple low state exposure increases [8–10]. Receiver operator curves (ROC) were generated for two models: 1) Median TVI value expressed during surgery and 2) total number of profile windows that met triple low state criteria during surgery. The areas under the curve (AUC) were compared using roc.test function in pROC package with the bootstrap method and 2000 bootstrapped samples.

Cardiac surgery is distinct from other types of surgical therapy, in part, because cardiopulmonary bypass (CPB) is commonly used. The effects of CPB are well-established [15] and may potentially influence TVI expression in ways not observed in other surgery types. To better understand TVI expression during cardiac surgery, cardiac surgery profiles with and without CPB were compared using the plotting method described above. In this plot, TVI profiles in each cluster were ordered according their associated procedure time (shortest first, longest last) so as to more clearly observe the CPB period. TVI values were compared between four surgical groups in each cluster: 1) All surgeries, 2) noncardiac surgery 3) cardiac surgery without CPB, and 4) cardiac surgery with CPB.

All above analyses were completed using RStudio Version 1.0.143 (https://www.R-project.org/).

## Results

### Study population

A total of 5296 surgeries were selected for study from 16,104 eligible surgeries that occurred during our study period. BIS and inhaled anesthetic concentrations were not recorded at any point in the electronic anesthetic record system in 10,576 and 7097 surgeries, respectively. Table 1 summarizes the patient and procedure characteristics associated with our study population prior to k-means clustering. A total of 333,179 MAP measurements, 168,007 BIS measurements, and 199,311 MAC measurements representing 4358 individual patients were available for TVI analysis. The most commonly assigned ASA Physical Status category was 3. Repeat and emergency surgery occurred in 17.7 and 10.4% of surgeries, respectively. Mean age at time of surgery was 53.6 years (SD 16.8), 53.8% patients were male, and median length of procedure was 1.8 h (Q1-Q3, 1.0–3.4). General, orthopedic, and thoracic surgery represent the three most common surgical specialties associated with our study population. Wound irrigation/debridement, exploratory laparotomy and anterior cervical spine discectomy with internal fusion/fixation were the three most common procedures performed.

**Table 1:** Patient, surgical, and TVI characteristics that define the study population

Variable	Study Population
Total Profiles	5296
Total Patients	4358
% Profiles representing repeat surgery	17.7
% ASA Physical Status 1	4.5
% ASA Physical Status 2	28.9
% ASA Physical Status 3	48.1
% ASA Physical Status 4	17.6
% ASA Physical Status 5	0.8
% Emergent Cases	10.4
Mean Age (SD)	53.6 (16.8)
% Male Gender	53.8
Most common surgical specialty (%)	1. General (12.8) 2. Orthopedic (8.9) 3. Thoracic (5.2)
Most common primary procedure (%)	1. Irrigation and Debridement of Wound (10.3) 2. Exploratory Laparotomy (5.1) 3. Anterior Spine Cervical Discectomy and Internal Fusion/Fixation (2.5)
Median Length of Procedure, hr. (Q1-Q3)	1.8 (1.0–3.4)
Total MAP measurements	333,179
Mean MAP, mmHg (SD)	81.4 (17.2)
Total BIS measurements	168,007
Mean BIS (SD)	41.3 (10.4)
Total MAC measurements	199,311
Mean MAC (SD)	0.81 (0.29)
Median frequency of MAP measurement, min (Q1-Q3)	3 (2–5)
Median frequency of BIS measurement, min (Q1-Q3)	5 (5–5)
Median frequency of MAC measurement, min (Q1-Q3)	5 (5–5)
Total TVI values	54,574
Mean TVI (SD) [min,max]	−0.09 (1.4) [− 8.1,7.5]
Median TVI values per profile (Q1-Q3) [min,max]	9 (5–13) [1,63]
Median proportion of missing TVI values due to CPB (Q1-Q3)	0.33 (0.24–0.40)
Median length of profile window, min (Q1-Q3)	10 (8–20)

The three most common surgical specialties and procedures associated with the population are shown. *SD* standard deviation, *Q1* 1st quartile, *Q3* 3rd quartile, *hr.* hour, *Min* minutes

**Fig. 1:**
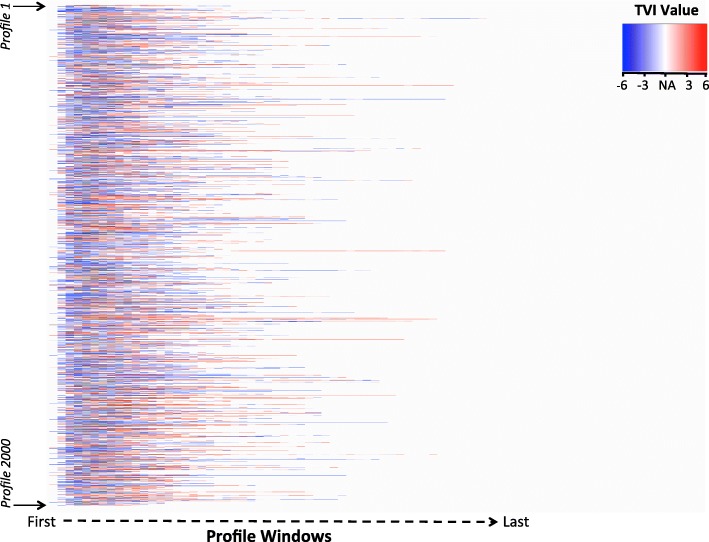
2000 randomly sampled TVI profiles from the study population. White color (NA) represents windows that do not contain a TVI value. Red represents a relatively elevated TVI value, whereas blue represents a relatively depressed TVI value. TVI = Triple Variable Index

MAP, BIS, and MAC values were recorded during surgery at a median frequency of once every 3, 5, and 5 min, respectively. Their mean levels (SD) observed during surgery were 81.4 (17.2) mmHg, 41.3 (10.4), and 0.81 (0.29). MAP, BIS, and MAC values were similar between all observed surgeries, defined as those that occurred during the study period, and those selected for TVI analysis (Additional file 1: Table S1). Additional file 2: Figure S1 shows how raw MAP, BIS, and MAC data, after artifact removal, were converted to z-scores and combined to generate TVI profiles. The mean TVI value was − 0.09 (SD 1.4), the median number of TVI values generated per surgery was 9 (Q1-Q3, 5–13). The median length, in minutes, of each profile window was 10 (Q1-Q3, 8–20). In surgeries where cardiopulmonary bypass was used, the median proportion of missing TVI values was 0.33 (Q1-Q3, 0.24–0.40). Fig. 1 shows the TVI values as expressed across the intraoperative monitoring period for 2000 individual, randomly-sampled profiles.

### TVI expression pattern identification using k-means clustering

**Fig. 2:**
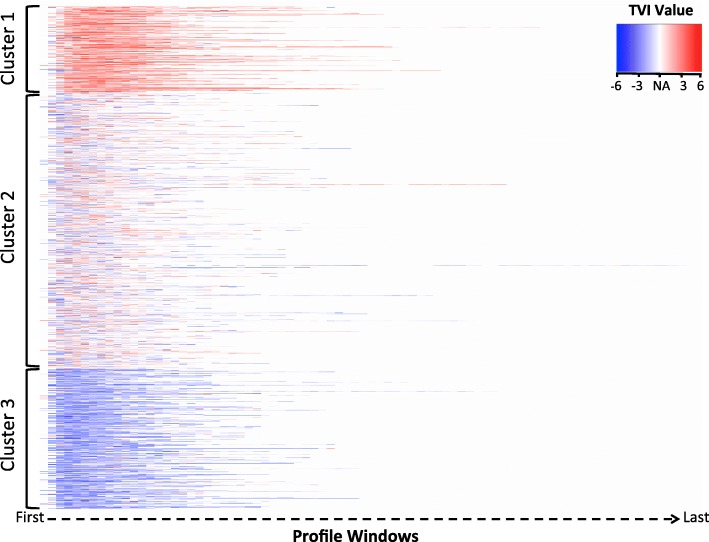
2000 sampled TVI profiles following k-means clustering where k = 3. White color (NA) represents windows that do not contain a TVI value. Red represents a relatively elevated TVI value, whereas blue represents a relatively depressed TVI value. TVI = Triple Variable Index

**Fig. 3:**
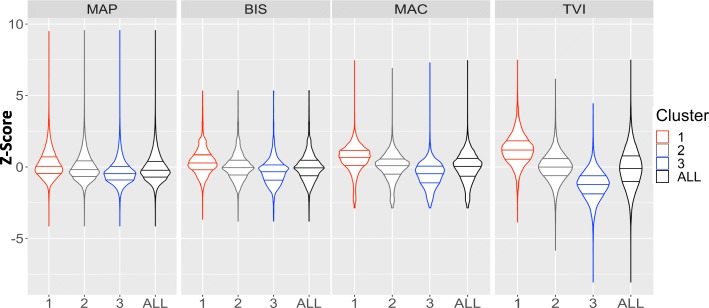
Violin plots showing the z-score distribution of each selected variable between the 3 clusters. MAP = Mean arterial pressure. BIS = Bispectral Index. MAC = Minimum alveolar concentration. TVI = Triple Variable Index

**Fig. 4:**
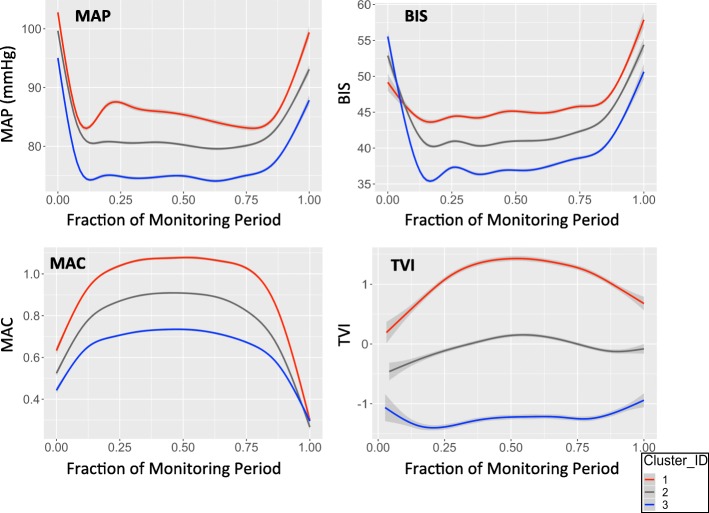
MAP, BIS, MAC, and TVI values as expressed across the intraoperative period for each cluster. These values are plotted across time as the proportion of the total monitoring period at which they occurred. Grey shading represents 95% confidence interval. MAP = Mean arterial pressure. BIS = Bispectral Index. MAC = Minimum alveolar concentration. TVI = Triple Variable Index

Figure 2 shows a random sample of 2000 individual TVI profiles following k-means clustering (k = 3). Profiles in cluster 1 demonstrated a consistent, relatively elevated TVI signal shown by the predominance of red across the profile windows. This can be compared to the relatively depressed TVI signal, shown as persistent blue, expressed in profiles identified in cluster 3. Cluster 2 profiles expressed a mixed, intermediate TVI signal, indicated by a mix of red and blue colors, also with lower intensity. MAP, BIS, MAC, and TVI z-score distributions for each cluster and the entire study population are shown in Fig. 3. MAP, BIS, MAC, and TVI values, as expressed across time in each cluster, are shown in Fig. 4. For the remainder of the manuscript, we define TVI expression patterns as follows: cluster 1 is elevated, cluster 2 is mixed, and cluster 3 is depressed.

### Characterization of elevated, mixed, and depressed TVI expression patterns

TVI patterns demonstrated distinct MAP, BIS, MAC, and TVI values (Table 2). For example, the mean MAP, BIS, MAC, and TVI z-scores for the elevated pattern were 0.22, 0.38, 0.56, and 1.21, respectively, while the corresponding z-scores in the depressed pattern were − 0.34, − 0.31, − 0.54, and − 1.25. Differences in these variables between expression patterns were observed across the majority of the intraoperative monitoring period (Fig. 4). BIS and MAC exhibited the highest correlations, elevated (− 0.43), mixed (− 0.45), and depressed (− 0.42), as shown in Additional file 3: Table S2. MAP-MAC and BIS-MAC correlations were higher within expression patterns compared to those calculated for the entire study population.

**Table 2:** Patient, surgical and TVI characteristics for each TVI expression pattern

Variable	Elevated TVI	Mixed TVI	Depressed TVI
Total Profiles	891	2931	1474
Total Patients	857	2554	1291
% Repeat surgery (95% CI)	3.8 (2.7–5.3)	12.9 (11.7–14.1)	12.4 (10.8–14.2)
% ASA Physical Status 1 (95% CI)	5.2 (3.8–6.9)	4.6 (3.9–5.5)	3.9 (3.0–5.1)
% ASA Physical Status 2 (95% CI)	36.8 (33.7–40.1)	29.6 (28.0–31.3)	22.7 (20.6–25.0)
% ASA Physical Status 3 (95% CI)	51.1 (47.8–54.5)	49.0 (47.2–50.9)	44.4 (41.9–47.0)
% ASA Physical Status 4 (95% CI)	6.7 (5.2–8.6)	16.1 (14.8–17.5)	27.3 (25.0–29.6)
% ASA Physical Status 5 (95% CI)	0.1 (0.0–0.7)	0.6 (0.3–0.9)	1.6 (1.1–2.5)
% Emergent Surgery (95% CI)	5.8 (4.4–7.6)	10.5 (9.5–11.7)	12.8 (11.2–14.7)
Mean Age (95% CI)	52.0 (50.9–53.1)	53.7 (53.1–54.4)	54.5 (53.6–55.3)
% Male Gender (95% CI)	56.9 (53.6–60.2)	53.4 (51.6–55.2)	52.7 (50.1–55.3)
Most common surgical specialty (%)	1. General (35) 2. Orthopedic (20) 3. Thoracic (11)	1. General (32) 2. Orthopedic (19) 3. Thoracic (10)	1. General (29) 2. Orthopedic (16) 3. Cardiac (16)
Most common primary procedure (%)	1. Irrigation and Debridement of Wound (5.5) 2. Exploratory Laparotomy (3.7) 3. Anterior Spine Cervical Discectomy and Internal Fusion/Fixation (3.6)	1. Irrigation and Debridement of Wound (11.9) 2. Exploratory Laparotomy (4.4) 3. Esophagus Dilatation (2.8)	1. Irrigation and Debridement of Wound (10.0) 2. Exploratory Laparotomy (7.4) 3. Aortic Valve Replacement/Repair (2.7)
Median Length of Procedure, hr. (95% CI)	2.5 (2.4–2.7)	1.4 (1.3–1.5)	2.0 (1.9–2.2)
Mean MAP, mmHg (95% CI)	86.5 (86.3–86.6)	82.3 (82.2–82.4)	76.6 (76.5–76.7)
Mean MAP, z-score (95% CI)	0.22 (0.22–0.23)	−0.02 (−0.02-(−0.01))	−0.34 (− 0.35-(− 0.33))
Mean BIS (95% CI)	45.3 (45.2–45.4)	41.8 (41.7–41.8)	38.0 (37.9–38.1)
Mean BIS, z-score (95% CI)	0.38 (0.37–0.39)	0.04 (0.03–0.05)	−0.31 (− 0.32-(− 0.31))
Mean MAC (95% CI)	0.980 (0.977–0.982)	0.817 (0.815–0.819)	0.666 (0.664–0.668)
Mean MAC, z-score (95% CI)	0.56 (0.55–0.56)	− 0.01 (− 0.02-(− 0.01))	0.54 (0.55-(− 0.53))
Mean TVI (95% CI)	1.21 (1.19–1.23)	0.01 (− 0.01–0.02)	−1.25 (−1.27-(−1.24))
Median TVI values per profile (Q1-Q3)	12 (9–18)	7 (4–11)	10 (6–14)

The three most common surgical specialties and procedures representing each pattern are displayed. *CI* Confidence Interval, *Q1* 1st quartile, *Q3* 3rd quartile, *hr* hour. Location: Line 357 in text

Compared to the elevated pattern, the depressed pattern was associated with a higher percentage of repeat and emergent surgery, ASA Physical Status 4 and 5 categories, and increased patient age at the time of surgery (Table 2). The mixed pattern was associated with these characteristics in an intermediate manner. For example, ASA Physical Status categories and patient age associated with the mixed pattern fall between the values observed in these variables in the elevated and depressed patterns.

Elevated and mixed patterns closely resemble each other in terms of associated surgical specialties and performed procedures. The depressed pattern was more frequently associated with cardiac surgery (Table 2, Additional file 4: Table S3), however, general and orthopedic specialties remained similarly represented in this pattern as in the others. The elevated pattern was associated with the longest median length of procedure, while the mixed pattern was associated with the shortest (2.5 vs 1.4 h).

**Fig. 5:**
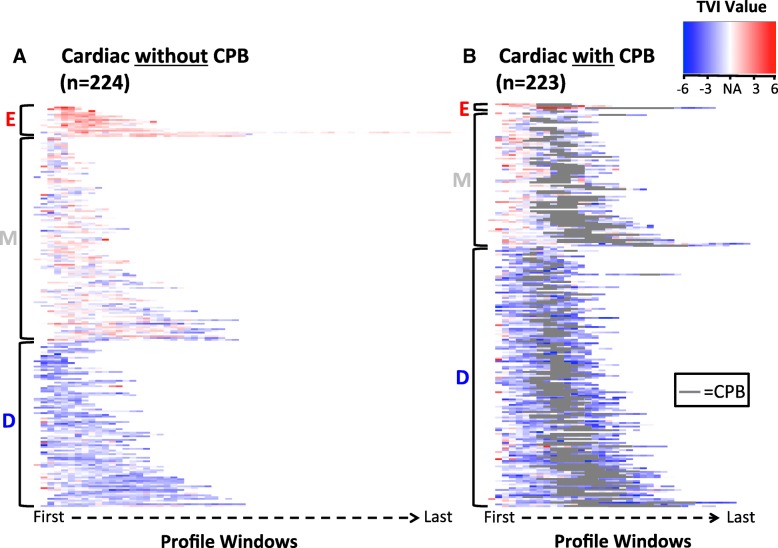
TVI expression associated with cardiac surgery. Red represents a relatively elevated TVI value, blue represents a relatively depressed TVI value, and white color (NA) represents windows that do not contain a TVI value. Panel A- Profiles where surgery was performed without the use of cardiopulmonary bypass (CPB). Panel B- Profiles where surgery was performed using CPB. For each TVI pattern in each plot, profiles were ordered from shortest to longest procedure times. The grey area in the CPB-associated profiles represents the CPB period. “E”, “M”, and “D” denote elevated, mixed and depressed TVI profiles. TVI = Triple Variable Index

**Fig. 6:**
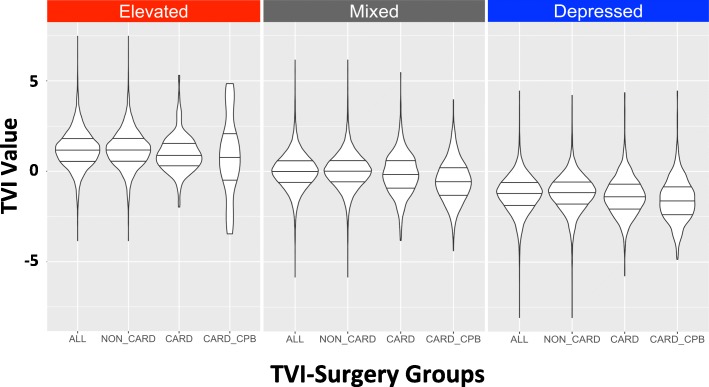
Violin plots showing the distribution of TVI values for defined surgery groups. For each pattern, TVI values were compared between all surgeries (ALL), noncardiac surgery (NON_CARD), cardiac surgery without cardiopulmonary bypass (CARD), and cardiac surgery with cardiopulmonary bypass (CARD_CPB). TVI = Triple Variable Index

Cardiac surgery represents 447 TVI profiles in the total study population, 223 of these employed CPB and 224 did not (Fig. 5). All three TVI patterns were observed in cardiac surgery independent of CPB use. Differences in TVI expression were observed between noncardiac, cardiac without CPB, and cardiac with CPB surgeries (Fig. 6 and Additional file 4: Table S3), however these differences were smaller than those observed between TVI patterns overall. The largest differences were observed between noncardiac and cardiac with CPB profiles; in the depressed pattern, for example, the mean TVI values were − 1.20 and − 1.60, respectively. Compare these values to those observed in cardiac surgery with CPB in each pattern: 0.83 (elevated), − 0.53 (mixed), and − 1.60 (depressed).

Common intravenous anesthetics, anesthetic adjuncts, opioids, vasopressors, and muscle relaxants administered during surgery for each pattern are shown Additional file 5: Table S4 and Additional file 6: Table S5. Defined as the proportion of profiles where a given medication was administered at least once, the elevated pattern was more commonly associated with hydromorphone (47.6%), ketamine (11.2%), dexmedetomidine (18.4%), and succinylcholine (74.0%) use compared to the depressed pattern. Conversely, the depressed pattern was more frequently associated with etomidate (12.6%), remifentanil (8.5%), phenylephrine (83.0%), epinephrine (18.0%), norepinephrine (21.2%), and vasopressin (21.0%) use compared to the elevated pattern. Propofol and fentanyl were administered almost universally and independent of TVI pattern (> 95% of all profiles). The median total dose of propofol administered in elevated pattern surgeries was 185 mg compared to 150 mg observed in depressed pattern surgeries (Additional file 6: Table S5). The median total dose of fentanyl administered was 250mcg for surgeries across all patterns.

Patterns were associated with different levels of intraoperative hypotension and triple low state exposure during surgery (Table 3). Less than 30% of profiles in the elevated pattern experienced intraoperative hypotension, defined as a MAP less than 55 mmHg, compared to more than half (52.3%) of the profiles in the depressed pattern. Differences in triple low state exposure were even more pronounced: 9.4% of profiles in the elevated pattern experienced the triple low state compared to 80.0% of those in the depressed pattern. Notably, these differences were not a function of blood pressure monitoring frequency or rate of TVI value generation.

**Table 3:** Intraoperative hypotension and triple low state exposure associated with TVI patterns

Variable	Elevated TVI	Mixed TVI	Depressed TVI
Total profiles	891	*2931*	1474
% Profiles experiencing IOH (95% CI)	28.8 (25.9–32.0)	*33.0 (31.3–34.8)*	52.3 (49.7–54.9)
% Profiles experiencing TLS (95% CI)	9.4 (7.6–11.6)	*30.3 (28.7–32.0)*	80.0 (77.8–82.0)
Median MAPs/hr. (Q1-Q3)	30 (19–36)	*33 (22–44)*	30 (18–41)
Median TVIs/hr. (Q1-Q3)	6 (3–7)	*6 (3–7)*	6 (3–7)

Adverse intraoperative events and postoperative death associated with TVI patterns. Table 3, Proportions of total profiles that experienced intraoperative hypotension (IOH) and triple low state (TLS) exposure for each pattern. Median rates of data capture for MAP and TVI values are shown. *Q1* 1st quartile, *Q3* 3rd quartile, *hr* hour

The postoperative mortality associated with each pattern is shown in Tables 4 and 5. In Table 4, 5.6% of profiles in the depressed pattern were associated with patients that died within 30 days following surgery. This is compared to 0.8 and 2.7% of profiles observed in the elevated and mixed patterns, respectively. Patterns do not exhibit a difference in their associated mortality between postoperative days 31 and 730. Postoperative mortality associated with the mixed and depressed patterns decreased the most in the sensitivity analysis (Table 5), consistent with our findings that these patterns are associated with the highest frequency of repeat surgery (Table 2). Recalculation of mortality proportions in the sensitivity analysis did not alter the overall trend observed in the study population. For example, the 30-day postoperative mortality observed in the elevated and depressed patterns was 0.8 and 5.6%, and 0.7 and 5.0% following the sensitivity analysis.

**Table 4:** Postoperative mortality as a proportion of total profiles in each pattern

Variable	Elevated TVI	Mixed TVI	Depressed TVI
% 30 Day Mortality (95% CI)	0.8 (0.3–1.7)	2.7 (2.2–3.4)	5.6 (4.5–6.9)
% 31–365 Day Mortality (95% CI)	4.9 (3.7–6.6)	6.7 (5.9–7.7)	7.4 (6.1–8.9)
% 366–730 Day Mortality (95% CI)	3.3 (2.2–4.7)	3.8 (3.1–4.5)	4.3 (3.4–5.5)

Adverse intraoperative events and postoperative death associated with TVI patterns. Table 4, Proportion of profiles in each pattern associated with postoperative death outcomes

**Table 5:** Postoperative mortality as a proportion of total individual patients in each pattern

Variable	Elevated TVI	Mixed TVI	Depressed TVI
% 30 Day Mortality (95% CI)	0.7 (0.3–1.6)	2.5 (2.0–3.2)	5.0 (3.9–6.3)
% 31–365 Day Mortality (95% CI)	5.0 (3.7–6.8)	6.4 (5.5–7.5)	7.0 (5.7–8.5)
% 366–730 Day Mortality (95% CI)	3.3 (2.2–4.7)	3.7 (3.0–4.5)	4.2 (3.2–5.5)

Adverse intraoperative events and postoperative death associated with TVI patterns. Table 5, Proportions re-calculated using only individual patients identified in each TVI pattern. Location: Line 410 in text

**Fig. 7:**
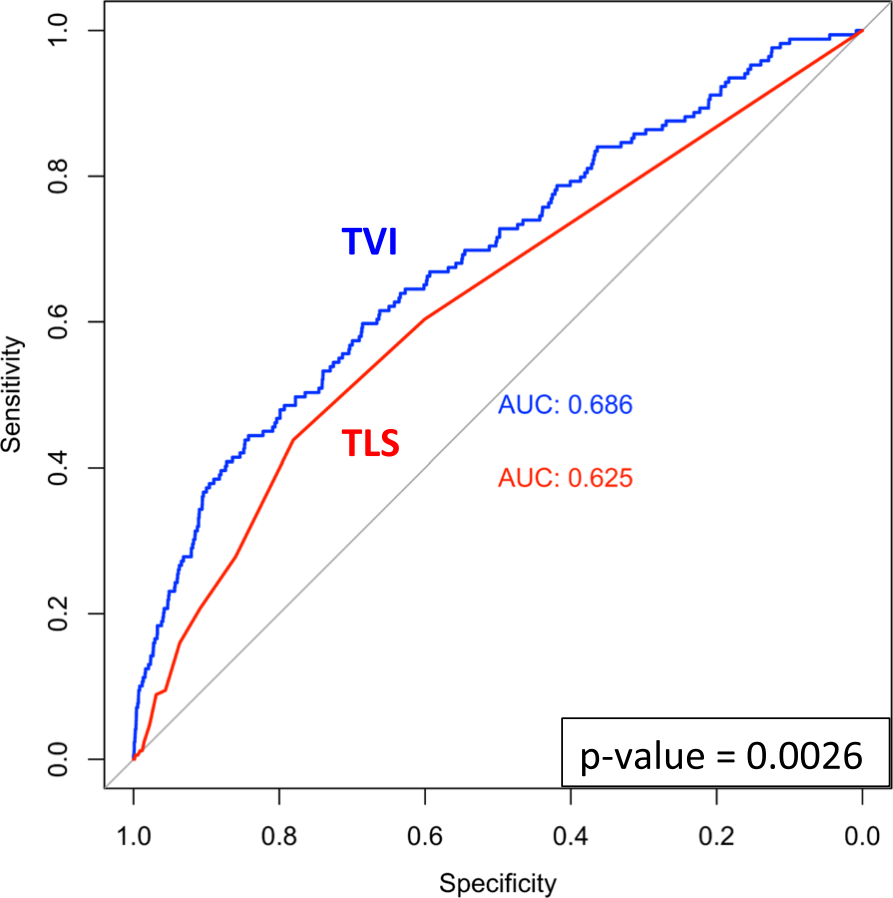
TVI and the triple low state compared as prediction models for 30-day postoperative death. The median TVI value for each surgery represented the TVI model; the total number of profile windows that met triple low state criteria (MAP < 75 mmHg, BIS < 45, MAC < 0.8) during surgery represented the triple low state model. ROC for each model is shown along with its associated AUC. *P*-value of the statistical test used to compare AUCs is shown in lower right corner. TVI = Triple Variable Index. TLS = triple low state

Figure 7 compares TVI expression and the triple low state as predictors of 30-day postoperative mortality. The median TVI value generated during surgery discriminated patients that died or survived the 30 days following surgery better than cumulative triple low state exposure (defined as the total number of profile windows that met triple low criteria during surgery). The AUC for the TVI model was 0.686 compared to 0.625 for the triple low state model. The difference between these values was statistically significant (*p*-value 0.002).

## Discussion

MAP, BIS, and MAC monitoring can provide valuable information about a patient’s risk of experiencing poor postoperative outcomes. We have created the Triple Variable Index that combines MAP, BIS, MAC data for any surgery in which they were generated and across time. From more than 5200 surgeries, 700,497 individual MAP, BIS, and MAC measurements were used to calculate and maps 54,574 TVI values across the intraoperative period. We identified and characterized three distinct patterns of TVI expression including their relationship to intraoperative adverse events and postoperative mortality.

Multiple patient and procedure-related factors characterize patterns of TVI expression and likely contribute to their distinct MAP, BIS, and MAC values. The depressed pattern, characterized by low MAP, BIS, and MAC values, was associated with increased patient age and higher ASA Physical Status categories. Increased age and disease burden (e.g. anemia) potentiate the effects of volatile anesthetics [11, 16]. Cardiopulmonary bypass has many known physiologic effects including hypothermia and vasoplegia [15, 17]; these alone or in combination with other factors appear to depress TVI expression. In both mixed and depressed patterns, lower TVI values were observed in cardiac surgery using CPB compared to noncardiac and cardiac surgery where CPB was not employed.

Intraoperative medications were differentially associated with patterns, however clear effects of single medications were not observed. For example, ketamine and muscle relaxants are associated with BIS levels unrelated to anesthetic depth [18, 19], while opioids and α_2_ agonists potentiate the effect of volatile anesthetics [20]. All of these medications were most frequently associated with the same pattern (elevated) despite potentially influencing TVI expression in distinct ways. Vasopressors decrease the vasodilating effect of volatile anesthetics through their direct mechanisms. Yet the depressed pattern, characterized by the lowest observed MAP-MAC combinations, was most frequently associated with these medications. It is possible patient co-morbidity and/or traumatic injury, reflected in the depressed pattern’s higher ASA Physical Status assignments and proportion of emergent surgeries, may explain this paradoxical association between low MAP levels and increased vasopressor use to some extent. Propofol, midazolam, fentanyl, and rocuronium were commonly administered and given at similar doses independent of TVI pattern. Remifentanil administration was most frequently associated with the depressed pattern, however it was used in less than 10% of surgeries.

It is important to consider how our clustering approach may have influenced our overall findings. The number of clusters selected for analysis is chosen a priori and those derived from analyses selecting a different k value (e.g. 4 or 5) may be associated with subtly different characteristics from those reported here. However, TVI represents an unadjusted measure of response to inhaled anesthetics and the characteristics associated with relatively low or high MAP and BIS values at a given MAC administration would be expected to be observed independent of the number of selected clusters. For example, higher ASA Physical Status assignments and increased patient age would be more likely associated with low rather than high TVI values given the known effects of age and disease burden on a patient’s response to inhaled anesthetics. Second, although TVI values for an individual surgery represent time-series data with some level of correlation, distinguishing profiles according to the pattern of repeating values expressed across time represents the basis of our approach. As a result, we did not attempt to adjust our analysis to account for such correlations. Finally, other approaches may be used to distinguish profiles in meaningful ways in addition to k-means clustering. Our prediction model for 30-day postoperative death, for instance, used the median TVI value generated during surgery.

Our study has several limitations. MAP, BIS, and MAC variables were given equal weights relative to the construction of the TVI variable. For predictive purposes, unequal weights may be more useful but remain unknown. It is possible to apply existing feature selection/extraction algorithms to identify the MAP, BIS, and MAC weights that better predict specific events like postoperative death [21]. To combine MAP, BIS, and MAC data, we used a sliding window approach based upon several assumptions including the calculated statistic within the window (mean) and the window’s size (five consecutive measurements). A median statistic would be more resistant to outlier values within the window than a mean, and as a result, may modestly reduce the amount of variation in TVI values within individual profiles. Increasing window length would have a similar effect. Although not tested directly, it is difficult to argue that either scenario would significantly affect the overall TVI differences observed between identified patterns. Our intraoperative medication analysis was limited to the proportion of profiles in each pattern that received a given medication during surgery and the median total dose administered. Without considering the pharmacokinetic and pharmacodynamic aspects of the medications and how they may differ between the surgeries, our findings must be carefully interpreted. The effect of individual medications is best evaluated using a controlled experimental design where patient weight, dosing, co-administered medications, for example, can be controlled between experimental groups. Future studies are needed to address these limitations.

Several key similarities and differences are important to note between the triple low state and our presented Triple Variable Index. Both leverage the information captured within concurrently monitored MAP, BIS, and MAC variables, however missing data were “carried over” from previous measurements or interpolated between measurements in two of the original triple low state studies [8, 9]. TVI makes no assumptions related to missing data and averaged data within defined windows of time similar to Willingham’s et al. epoch approach [10]. TVI used non-age adjusted MAC values because the goal was to evaluate variable combinations as they are measured without adjusting for factors known to affect the underlying relationships between the variables. This was the most common approach used in the triple low state studies [9, 10]. Using age-adjusted MAC values would likely reduce the difference in TVI values observed between patterns because MAC values would become relatively larger for given MAP and BIS values as age increases. Our approach to missing data and MAC calculations highlights our attempt to make few assumptions about the existing data and represents a key aspect of TVI as a data-driven index. Our study represents the first to evaluate MAP, BIS, and MAC combinations in any adult study patient with requisite data and without using variable thresholds, plot the data in a temporal fashion, evaluate intraoperative medication administration across multiple drug classes, and assess intraoperative hypotension (IOH) exposure, an important adverse event linked to end organ damage and postoperative death [22].

TVI better discriminated patients that died or survived in the 30 days following surgery compared to cumulative triple low state exposure in our study population. However, the utility of TVI as a predictor of postoperative death is limited considering the predictive ability of previously established models such as the Risk Stratification Index (RSI) [23], Risk Quantification Index (RQI) [24], and a recently developed deep neural network [25]. TVI may be more useful as a tool to further explore the association between a patient’s response to inhaled anesthetics and IOH. Despite receiving approximately 30% less inhaled anesthetics, patients in the depressed pattern achieved an average MAP that was 10 points lower than those of the elevated pattern (76 versus 86 mmHg) leading to a 1.8 fold increase in IOH exposure. It remains unclear how patterns differ in terms of IOH frequency, location, and depth; all features that directly impact IOH-related complications and/or provide useful information relevant to predicting events. Two IOH prediction models have recently been developed but are limited to only patients with arterial line monitoring [26] or predicting IOH within a narrow window of time (i.e. post-induction) [27]. Leveraging TVI to further characterize IOH events will provide key information that may allows clinicians in the future to better predict and prevent their occurrence during surgery.

## Conclusions

Each time MAP, BIS, and inhaled anesthetic concentrations are measured together during surgery, they reveal key information about the patient that goes beyond appropriate blood pressure and anesthetic depth levels. TVI expression demonstrates these data, iteratively captured over time, form a pattern of physiology that reflects important patient, procedure, and outcome characteristics.

## Additional files

Additional file 1: **Table S1.** Mean MAP, BIS, and MAC values within observed and study (TVI) populations. SD = standard deviation. MAP = Mean arterial pressure. BIS = Bispectral Index. MAC = Minimum alveolar concentration. (PDF 30 kb)

Additional file 2: **Figure S1.** Schematic for converting MAP, BIS, and MAC data (after artifact removal) into TVI profiles. The numbers denote the general processing steps that take place: 1) Z-scores are calculated for each MAP, BIS, and MAC value. Values for each study surgery are labeled with a profile window number. The first five measurement timepoints represent the first profile window, the next five represent the second window, etc. 2) Average MAP, BIS, and MAC values are calculated for each profile window and a TVI value is generated by summing the averages if an average value exists for each variable. 3) The TVI values in sequential profiles widows represent a study surgery’s TVI profile. 4) Profiles can be plotted together and compared. TVI = Triple Variable Index. (PDF 1998 kb)

Additional file 3: **Table S2.** Pearson correlations between MAP, BIS, and MAC variables in all study profiles and those associated with elevated, mixed, and depressed TVI patterns. MAP = Mean arterial pressure. BIS = Bispectral Index. MAC = Minimum alveolar concentration. (PDF 30 kb)

Additional file 4: **Table S3.** TVI expression associated with non-cardiac and cardiac surgery. Proportion of TVI profiles associated with each surgical group and their mean TVI values. CI = Confidence Interval. CPB = Cardiopulmonary bypass. (PDF 33 kb)

Additional file 5: **Table S4.** Proportions of common intravenous anesthetics/adjuncts, opioids, vasopressors and muscle relaxants administered between TVI patterns. CI=Confidence Interval. (PDF 35 kb)

Additional file 6: **Table S5.** Median total dose of common intravenous anesthetics/adjuncts, opioids, vasopressors and muscle relaxants administered between TVI patterns. Q1 = 1st quartile, Q3 = 3rd quartile. (PDF 36 kb)

## Abbreviations

ASA: American Society of Anesthesiologists
AUC: Area under the curve
BIS: Bispectral Index
CPB: Cardiopulmonary bypass
IOH: Intraoperative hypotension
MAC: Minimum alveolar concentration
MAP: Mean arterial pressure
ROC: Receiver operating curve
TLS: Triple Low State
TVI: Triple Variable Index
