# Supplementary material for: The triple variable index combines information generated over time from common monitoring variables to identify patients expressing distinct patterns of intraoperative physiology
Source: BMC Med Res Methodol. 2019 Jan 14;19:17. doi: 10.1186/s12874-019-0660-9 (PMC6332613; doi:10.1186/s12874-019-0660-9)
Supplement: Supplementary file 1 — Table S1. Mean MAP, BIS, and MAC values within observed and study (TVI) populations. SD = standard deviation. MAP = Mean arterial pressure. BIS = Bispectral Index. MAC = Minimum alveolar concentration. (PDF 30 kb) [file 12874_2019_660_MOESM1_ESM.pdf]

Variable	Observed Population	TVI Population
Total Surgeries	16104	5296
Mean MAP, mmHg (SD)	82.6 (17.5)	81.5 (17.2)
Mean BIS (SD)	41.3 (10.6)	41.3 (10.4)
Mean MAC (SD)	0.821 (0.29)	0.808 (0.29)

Additional Table 1.
