# Supplementary material for: The triple variable index combines information generated over time from common monitoring variables to identify patients expressing distinct patterns of intraoperative physiology
Source: BMC Med Res Methodol. 2019 Jan 14;19:17. doi: 10.1186/s12874-019-0660-9 (PMC6332613; doi:10.1186/s12874-019-0660-9)
Supplement: Supplementary file 2 — Figure S1. Schematic for converting MAP, BIS, and MAC data (after artifact removal) into TVI profiles. The numbers denote the general processing steps that take place: 1) Z-scores are calculated for each MAP, BIS, and MAC value. Values for each study surgery are labeled with a profile window number. The first five measurement timepoints represent the first profile window, the next five represent the second window, etc. 2) Average MAP, BIS, and MAC values are calculated for each profile window and a TVI value is generated by summing the averages if an average value exists for each variable. 3) The TVI values in sequential profiles widows represent a study surgery’s TVI profile. 4) Profiles can be plotted together and compared. TVI = Triple Variable Index. (PDF 1998 kb) [file 12874_2019_660_MOESM2_ESM.pdf]

1. Surgery ID — Raw Data — Z-Scores — Profile Window

OR.Case.Number	Time	BIS	MAP	MAC	BIS_Z	MAP_Z	MAC_Z	Window_Number
XXX_2013_12345	01/22/14 07:54	NA	NA	NA	NA	NA	NA	1
XXX_2013_12345	01/22/14 07:55	NA	NA	NA	NA	NA	NA	1
XXX_2013_12345	01/22/14 07:56	NA	108	NA	NA	1.451	NA	1
XXX_2013_12345	01/22/14 07:58	NA	88	NA	NA	0.311	NA	1
XXX_2013_12345	01/22/14 08:00	NA	NA	NA	NA	NA	NA	1
XXX_2013_12345	01/22/14 08:01	NA	68	NA	NA	-0.83	NA	2
XXX_2013_12345	01/22/14 08:03	NA	133	NA	NA	2.877	NA	2
XXX_2013_12345	01/22/14 08:04	NA	113	NA	NA	1.737	NA	2
XXX_2013_12345	01/22/14 08:05	NA	NA	0.328	NA	NA	-1.724	2
XXX_2013_12345	01/22/14 08:06	NA	92	NA	NA	0.539	NA	2
XXX_2013_12345	01/22/14 08:08	NA	88	NA	NA	0.311	NA	3
XXX_2013_12345	01/22/14 08:10	NA	92	0.367	NA	0.539	-1.588	3
XXX_2013_12345	01/22/14 08:13	NA	75	NA	NA	-0.431	NA	3
XXX_2013_12345	01/22/14 08:15	66	101	0.384	2.326	1.052	-1.53	3
XXX_2013_12345	01/22/14 08:18	NA	105	NA	NA	1.28	NA	3
XXX_2013_12345	01/22/14 08:20	45.2	77	0.378	0.365	-0.317	-1.549	4
XXX_2013_12345	01/22/14 08:24	NA	85	NA	NA	0.14	NA	4
XXX_2013_12345	01/22/14 08:25	45	NA	0.961	0.346	NA	0.493	4
XXX_2013_12345	01/22/14 08:26	NA	76	NA	NA	-0.374	NA	4
XXX_2013_12345	01/22/14 08:28	NA	79	NA	NA	-0.203	NA	4

2. Mean Z-scores within each profile window — Sum of mean Z-scores

OR.Case.Number	Window_Number	BIS_Z_Aver	MAP_Z_Aver	MAC_Z_Aver	TVI_Value
XXX_2013_12345	1	NA	0.881	NA	NA
XXX_2013_12345	2	NA	1.081	-1.724	NA
XXX_2013_12345	3	2.326	0.550	-1.559	1.317
XXX_2013_12345	4	0.356	-0.188	-0.528	-0.361

3. TVI Profile for surgery XXX_2013_12345

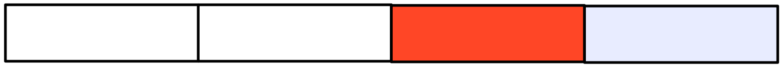

Window #	1	2	3	4
TVI Value	NA	NA	1.317	-0.361

4. Figure 2

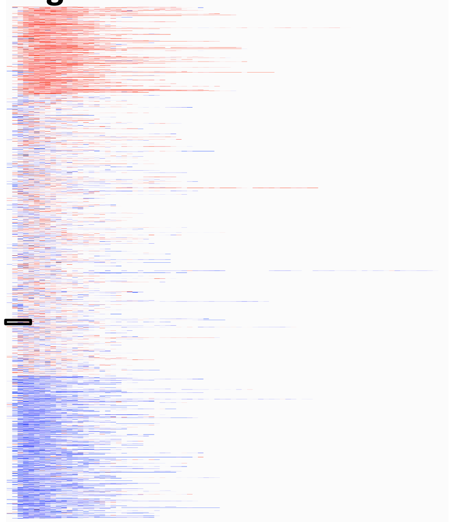

Additional Figure 1.
