# Supplementary material for: The triple variable index combines information generated over time from common monitoring variables to identify patients expressing distinct patterns of intraoperative physiology
Source: BMC Med Res Methodol. 2019 Jan 14;19:17. doi: 10.1186/s12874-019-0660-9 (PMC6332613; doi:10.1186/s12874-019-0660-9)
Supplement: Supplementary file 3 — Table S2. Pearson correlations between MAP, BIS, and MAC variables in all study profiles and those associated with elevated, mixed, and depressed TVI patterns. MAP = Mean arterial pressure. BIS = Bispectral Index. MAC = Minimum alveolar concentration. (PDF 30 kb) [file 12874_2019_660_MOESM3_ESM.pdf]

Variable	ALL Profiles	Elevated TVI	Mixed TVI	Depressed TVI
Total profiles	5296	891	2931	1474
MAP-MAC	-0.09	-0.20	-0.23	-0.13
BIS-MAC	-0.24	-0.43	-0.45	-0.42
MAP-BIS	0.07	-0.09	0.00	0.02

Additional Table 2.
