# Supplementary material for: The triple variable index combines information generated over time from common monitoring variables to identify patients expressing distinct patterns of intraoperative physiology
Source: BMC Med Res Methodol. 2019 Jan 14;19:17. doi: 10.1186/s12874-019-0660-9 (PMC6332613; doi:10.1186/s12874-019-0660-9)
Supplement: Supplementary file 4 — Table S3. TVI expression associated with non-cardiac and cardiac surgery. Proportion of TVI profiles associated with each surgical group and their mean TVI values. CI = Confidence Interval. CPB = Cardiopulmonary bypass. (PDF 33 kb) [file 12874_2019_660_MOESM4_ESM.pdf]

Variable	Elevated TVI	Mixed TVI	Depressed TVI
Total profiles	891	2931	1474
% Non-cardiac surgery (95% CI)	97.8 (96.5-98.6)	93.5 (92.5-94.4)	83.9 (81.9-85.7)
% Cardiac surgery without CPB (95% CI)	1.9 (1.2-3.1)	3.9 (3.2-4.7)	6.3 (5.1-7.7)
% Cardiac surgery with CPB (95% CI)	0.3 (0.1-1.1)	2.6 (2.1-3.3)	9.8 (8.3-11.4)
All surgeries- Mean TVI (95% CI)	1.21 (1.19-1.23)	0.01 (-0.01-0.02)	-1.25 (-1.27-(-1.24))
Non-cardiac surgery-Mean TVI (95% CI)	1.22 (1.20-1.23)	0.03 (0.02-0.04)	-1.20 (-1.22-(-1.19)
Cardiac surgery without CPB- Mean TVI (95% CI)	0.98 (0.85-1.10)	-0.17 (-0.25-(-0.10))	-1.36 (-1.43-(-1.30))
Cardiac surgery with CPB- Mean TVI (95% CI)	0.83 (0.06-1.59)	-0.53 (-0.62-(-0.45))	-1.60 (-1.66-(-1.54))

Additional Table 3.
