# Supplementary material for: The triple variable index combines information generated over time from common monitoring variables to identify patients expressing distinct patterns of intraoperative physiology
Source: BMC Med Res Methodol. 2019 Jan 14;19:17. doi: 10.1186/s12874-019-0660-9 (PMC6332613; doi:10.1186/s12874-019-0660-9)
Supplement: Supplementary file 5 — Table S4. Proportions of common intravenous anesthetics/adjuncts, opioids, vasopressors and muscle relaxants administered between TVI patterns. CI=Confidence Interval. (PDF 35 kb) [file 12874_2019_660_MOESM5_ESM.pdf]

Variable	Elevated TVI	Mixed TVI	Depressed TVI
Total Profiles	891	2931	1474
% Midazolam (95% CI)	86.2 (83.7-88.4)	81.7 (80.2-83.1)	83.0 (81.0-84.9)
% Propofol (95% CI)	99.7 (98.9-99.9)	99.0 (98.6-99.3)	98.1 (97.2-98.7)
% Etomidate (95% CI)	3.0 (2.0-4.4)	7.3 (6.4-8.3)	12.6 (10.9-14.4)
% Fentanyl (95% CI)	98.2 (97.0-98.9)	97.1 (96.4-97.6)	97.4 (96.4-98.1)
% Remifentanil (95% CI)	5.2 (3.8-6.9)	6.1 (5.3-7.1)	8.5 (7.1-10.0)
% Hydromorphone (95% CI)	47.6 (44.3-50.9)	26.3 (24.8-28.0)	25.2 (23.1-27.6)
% Morphine (95% CI)	1.3 (1.0-2.4)	1.1 (0.8-1.6)	0.9 (0.5-1.5)
% Ketamine (95% CI)	11.2 (9.3-13.5)	4.6 (3.9-5.4)	3.0 (2.2-4.0)
% Dexmedetomidine (95% CI)	18.4 (15.9-21.1)	16.6 (15.3-18.0)	13.6 (11.9-15.4)
% Ephedrine (95% CI)	61.5 (58.2-64.7)	58.9 (57.0-60.6)	59.2 (56.7-61.7)
% Phenylephrine (95% CI)	74.0 (70.9-76.8)	74.8 (73.2-76.3)	83.0 (81.0-84.9)
% Epinephrine (95% CI)	2.0 (1.2-3.2)	6.0 (5.2-7.0)	18.0 (16.1-20.1)
% Norepinephrine (95% CI)	2.4 (1.5-3.6)	7.5 (6.6-8.6)	21.2 (19.2-23.4)
% Vasopressin (95% CI)	3.4 (2.3-4.8)	7.6 (6.7-8.6)	21.0 (19.0-23.2)
% Succinylcholine (95% CI)	74.0 (70.9-76.8)	69.0 (67.3-70.7)	61.9 (59.4-64-4)
% Rocuronium (95% CI)	90.2 (88.0-92.1)	86.1 (84.8-87.3)	88.7 (86.9-90.2)
% Cisatracurium (95% CI)	4.2 (3.0-5.7)	3.8 (3.2-4.6)	5.7 (4.6-7.0)

Additional Table 4.
