# Supplementary material for: The triple variable index combines information generated over time from common monitoring variables to identify patients expressing distinct patterns of intraoperative physiology
Source: BMC Med Res Methodol. 2019 Jan 14;19:17. doi: 10.1186/s12874-019-0660-9 (PMC6332613; doi:10.1186/s12874-019-0660-9)
Supplement: Supplementary file 6 — Table S5. Median total dose of common intravenous anesthetics/adjuncts, opioids, vasopressors and muscle relaxants administered between TVI patterns. Q1 = 1st quartile, Q3 = 3rd quartile. (PDF 36 kb) [file 12874_2019_660_MOESM6_ESM.pdf]

Variable	Elevated TVI	Mixed TVI	Depressed TVI
Midazolam, mg (Q1-Q3)	2 (2-2)	2 (2-2)	2 (2-2)
Propofol, mg (Q1-Q3)	185 (143-200)	160 (120-200)	150 (109-200)
Etomidate, mg (Q1-Q3)	20 (19-24)	20 (16-24)	20 (14-20)
Fentanyl, mcg (Q1-Q3)	250 (250-450)	250 (150-300)	250 (150-400)
Remifentanil, mcg (Q1-Q3)	3769 (2103-5791)	3199 (1864-4959)	3025 (1615-4452)
Hydromorphone, mg (Q1-Q3)	2.0 (1.0-3.2)	1.6 (1.0-2.8)	1.6 (1.0-3.0)
Morphine, mg (Q1-Q3)	10 (5-14)	6 (4-10)	7 (4-10)
Ketamine, mg (Q1-Q3)	50 (30-70)	30 (20-50)	50 (30-60)
Dexmedetomidine, mcg (Q1-Q3)	28 (16-40)	30 (20-40)	32 (19-40)
Ephedrine, mg (Q1-Q3)	10 (0-25)	10 (0-20)	10 (0-20)
Phenylephrine, mcg (Q1-Q3)	400 (80-1520)	400 (80-1120)	480 (160-1360)
Epinephrine, mcg (Q1-Q3)	45 (20-198)	173 (25-594)	447 (60-1002)
Norepinephrine, mcg (Q1-Q3)	850 (265-1852)	493 (217-1279)	664 (237-1597)
Vasopressin, Units (Q1-Q3)	4 (2-8)	4 (2-8)	6 (3-13)
Succinylcholine, mg (Q1-Q3)	160 (140-200)	160 (120-200)	140 (120-200)
Rocuronium, mg (Q1-Q3)	85 (50-130)	60 (35-100)	80 (50-130)
Cisatracurium, mg (Q1-Q3)	30 (14-40)	20 (10-31)	24 (14-40)

Additional Table 5.
